# Severe acute respiratory syndrome coronavirus 2, primary varicella zoster virus coinfection, and a polymicrobial ventilator-associated tracheobronchitis in an adult immunocompetent male: a case report

**DOI:** 10.1186/s13256-022-03253-6

**Published:** 2022-01-24

**Authors:** Jowita Bruno, Silvio Ragozzino, Jonas Quitt, Martin Siegemund, Niklaus Labhardt

**Affiliations:** 1 Department of Intensive Care, University Hospital Basel, Petersgraben 4, 4031 Basel, Switzerland; 2 Department of Infectious Diseases, University Hospital Basel, Spitalstrasse 21, 4031 Basel, Switzerland; 3 Department of Medicine, Swiss Tropical and Public Health Institute, Basel, Switzerland; 4 University of Basel, Basel, Switzerland

**Keywords:** SARS-CoV-2, COVID-19, COVID-19-related skin lesions, Varicella zoster virus, Chickenpox, Coinfection

## Abstract

**Background:**

The spectrum of clinical manifestations and differential diagnosis associated with coronavirus disease 2019 is broad, ranging from fever and cutaneous eruptions to respiratory distress or even neurological disorders. Coexisting multipathogen infections significantly increase the complexity of the proper diagnostic and therapeutic approach and correlate with the rate of intensive care unit admissions and in-hospital mortality.

**Case presentation:**

We present a case of multipathogen respiratory infection with severe acute respiratory syndrome coronavirus 2, varicella zoster virus, and polymicrobial tracheobronchitis in a 48-year-old Caucasian male hospitalized after traumatic brain injury. The patient tested positive for severe acute respiratory syndrome coronavirus 2 infection upon admission. During his stay in the intensive care unit, the patient developed a vesicular exanthema along with respiratory failure and signs of septic shock.

**Conclusion:**

This case of an adult presenting with severe acute respiratory syndrome coronavirus 2 infection and simultaneous primary varicella zoster virus infection illustrates the importance of considering coinfections in patients with coronavirus disease 2019 with unusual clinical manifestations.

## Introduction

The spectrum of clinical manifestations and differential diagnosis associated with coronavirus disease 2019 (COVID-19) is broad, ranging from fever and cutaneous eruptions to respiratory distress or even neurological disorders. Coinfections and superinfections significantly increase the complexity of the proper diagnostic and therapeutic approach. Patients with coinfections seem to have higher rates of intensive care unit (ICU) admission, while those with hospital-acquired superinfections have longer hospital stays and higher mortality [1]. A potential immunosuppressive effect of severe acute respiratory syndrome coronavirus 2 (SARS-CoV-2), the virus causing COVID-19, infection potentially increases the risk of coinfections and influences their severity [2–5]. Here we describe a case of an uncommon coinfection in a patient with COVID-19 and highlight the importance of considering concomitant infectious processes in case of unusual manifestations.

## Case report

A 48-year-old Caucasian male with traumatic epidural hematoma and subarachnoid bleeding was transferred, after rapid neurological deterioration necessitating intubation, from another hospital to a tertiary university hospital for neurosurgical intervention. The patient had a history of preexisting fever and nonproductive cough over a period of 3–4 days prior to admission and had direct contact with a person who tested positive for SARS-CoV-2. A SARS-CoV-2 polymerase chain reaction (PCR) test performed upon admission was positive. The patient had no preexisting comorbidities, medications, or interventions.

Due to the positive SARS-CoV-2 swab, further management was performed according to the then valid institutional guidelines. Directly after the admission to our hospital, an uncomplicated emergency craniotomy for evacuation of the epidural hematoma and insertion of an intraparenchymal intracranial pressure (ICP) probe was performed, after which the patient was transferred to the ICU. Seventy-two hours after the admission to the ICU, a decompressive craniectomy was necessary due to edema of the left hemisphere with a midline shift of 6 mm. Further ICP management was uncomplicated. On the day of the second intervention, the patient developed a maculopapular erythematous exanthema, which progressed to a papulovesicular rash within 24–48 hours. Simultaneous to the progression of cutaneous eruptions, the patient developed respiratory failure and signs of septic shock.

### Physical examination

**Fig. 1:**
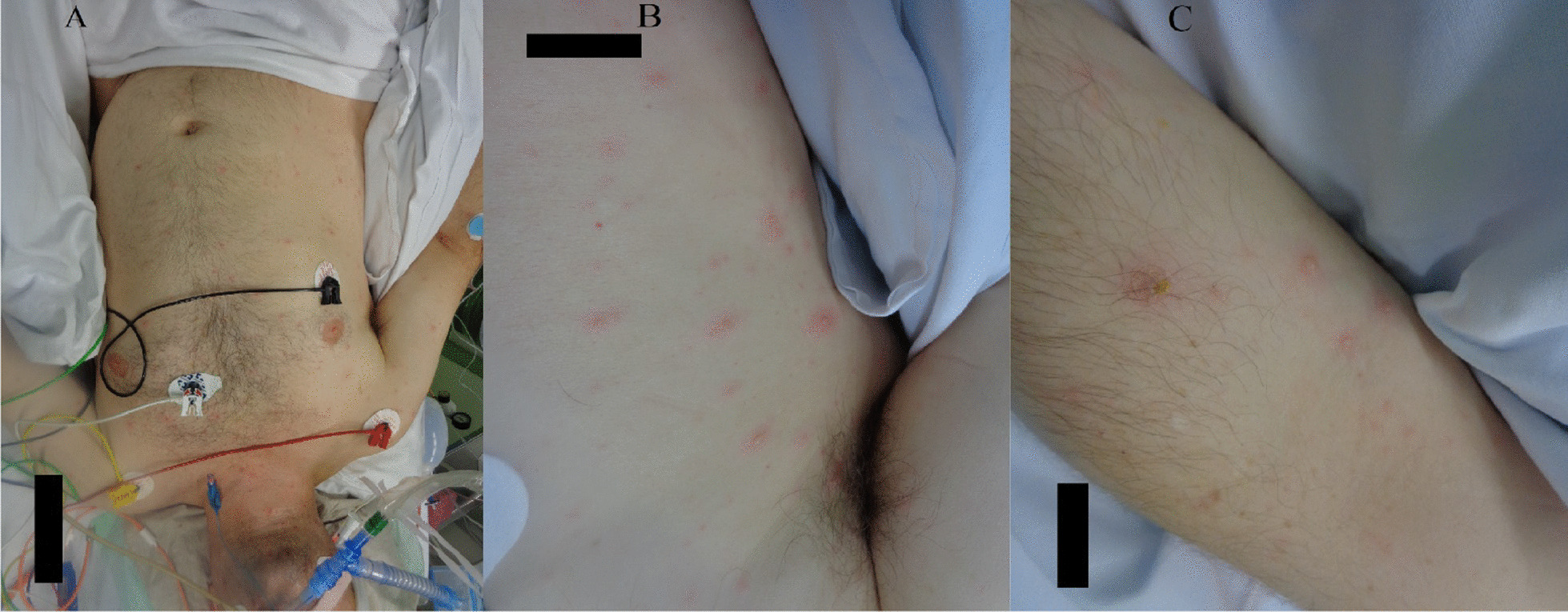
**A**–**C** Papulovesicular cutaneous eruptions involving trunk and extremities

Upon admission to the ICU, the patient presented with mild hypotension (95/60 mmHg) and a normal heart rate of 63 beats per minute. The patient was fully sedated with propofol and fentanyl [Glasgow coma scale (GCS) score of 3 points], intubated, and volume-controlled ventilated with a fractional concentration of inspired oxygen (FiO_2_) of 0.55, positive end expiratory pressure (PEEP) of 7 mbar, and respiratory rate of 17 breaths per minute. His body temperature was 39 °C. About 72 hours after the admission, a maculopapular erythematous exanthema predominantly on the trunk and upper and lower extremities was noted. Mucosae, palmar surfaces of hands, and plantar surfaces of feet were spared (Fig. 1). The exanthema was initially interpreted as a possible cutaneous manifestation of the SARS-CoV-2 infection. However, over the following days the exanthema progressed to a papulovesicular rash. The patient developed severe septic shock requiring volume substitution and high-dose vasoactive agents, as well as progressive respiratory failure with rapidly rising oxygen demand.

### Diagnostics

Routine laboratory tests upon admission were unremarkable except for slightly increased inflammatory markers and lymphopenia. Blood gas analysis was normal with an oxygenation index of 490 (FiO_2_ 0.55, partial pressure of oxygen in arterial blood 272 mmHg), and an initial thoracic computed tomography (CT) scan revealed no pulmonary infiltrates. Along with clinical signs of septic shock, inflammatory markers increased, and oxygenation index decreased (Fig. 3a, b).

**Fig. 2:**
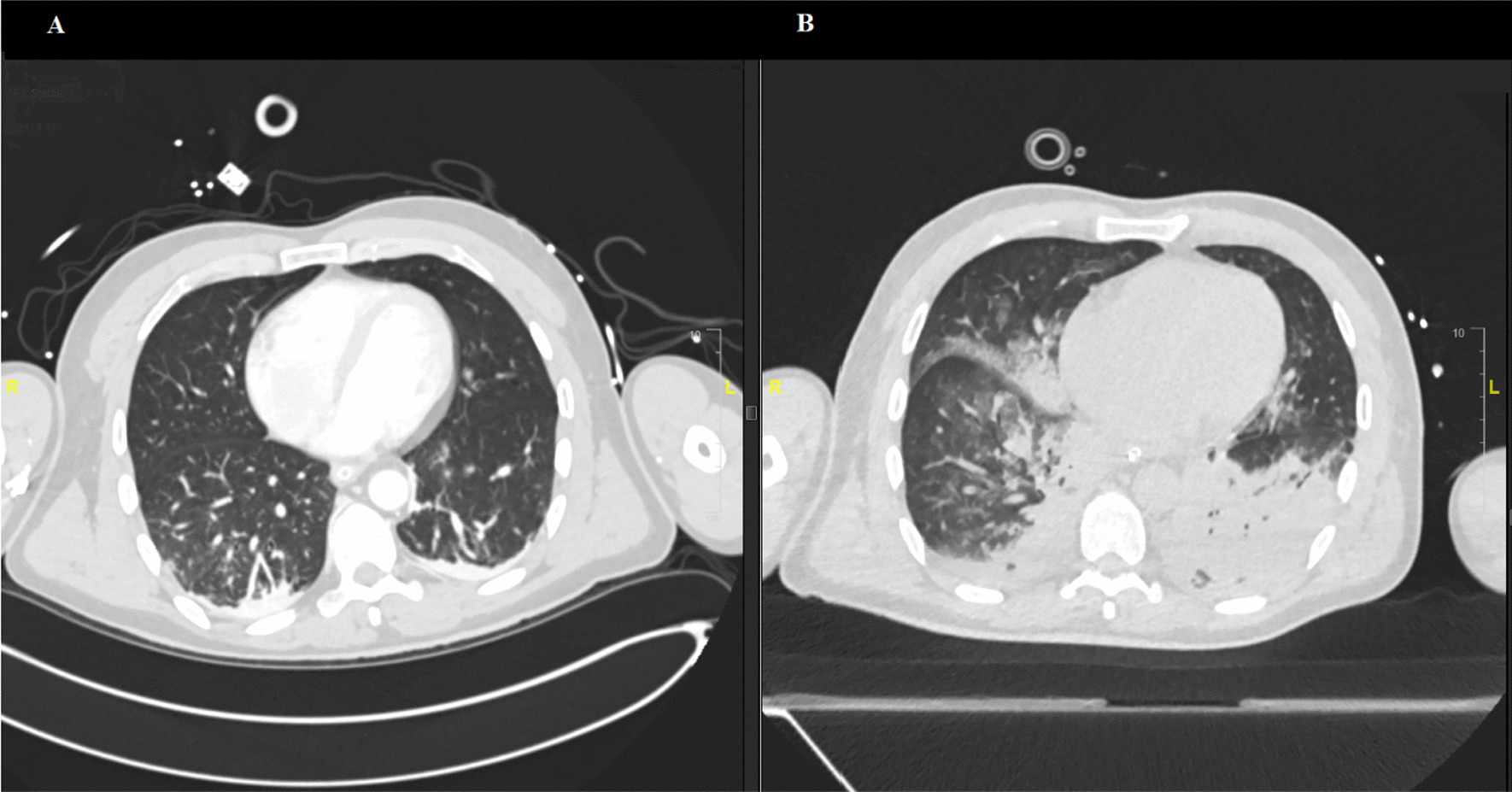
Computed tomography scans of the chest (**A**) on admission showing no signs of pulmonary infection (**B**) on the fifth day of stay in the ICU: patchy ground-glass opacifications and progressive consolidations typical for COVID-19 along with a small bilateral pleural effusions

A second CT scan performed on the fifth day after the admission revealed patchy ground-glass opacifications and progressive consolidations typical for COVID-19 present in 23% of the lung tissue volume. Small bilateral pleural effusions were also described (Fig. 2). The severe hemodynamic and respiratory deterioration (oxygenation index at this point was 97) was suggestive of a bacterial coinfection. However, because of the cutaneous manifestations, varicella zoster virus (VZV) was also considered. A PCR from a swab taken from a cutaneous vesicular lesion was positive for VZV and negative for herpes simplex virus types 1–2 and SARS-CoV-2. Serological testing confirmed primary VZV infection (Table 1). A bedside bronchoscopy was performed. Microbiological testing of the tracheal secretion revealed a polymicrobial bacterial flora with *Staphylococcus aureus*, *Enterobacter cloacae*, and *Serratia marcescens*. In addition, a specific PCR test was positive for VZV (4700 GEq/ml). Acute encephalitis, a possible complication of the surgeries, was also taken into account. Bacterial culture of the cerebrospinal fluid and multiplex PCR panel including VZV were negative.

**Table 1:** Laboratory test upon ICU admission (day 0) and on day 4

Value, unit (normal range)	Day 0	Day 4
White blood cells × 10^9^/l (3.5–10.0)	10.4	11.8
Lymphocytes × 10^9^/l (0.9–3.30)	0.5	1.3
Lactate, mmol/l (< 1.8)	0.9	2.1
VZV IgM, index (negative < 1.0)	–	> 2.3
VZV IgG, mlU/ml (negative < 150)	–	61

VZV IgM: Immunglobulin M to Varicella Zoster Virus; VZV IgG: Immunglobulin G to Varicella Zoster Virus

### Diagnosis

In addition to the COVID-19 pneumonia, a primary VZV coinfection with possible pulmonary involvement and ventilator-associated multibacterial tracheobronchitis with *Staphylococcus aureus*, *E. cloacae*, and *Serratia marcescens* were diagnosed. Due to rapid, progressive hemodynamic and respiratory deterioration, COVID-19 treatment with remdesivir (5 days) and dexamethasone (10 days) according to the then valid institutional protocol for COVID-19 management was started. Simultaneously, a broad-spectrum antibiotic coverage with meropenem (for 7 days) was initiated, and the primary VZV infection with possible pulmonary involvement was treated with high-dose acyclovir (10 mg/kg every 8 hours for 14 days).

### Clinical course

**Fig. 3:**
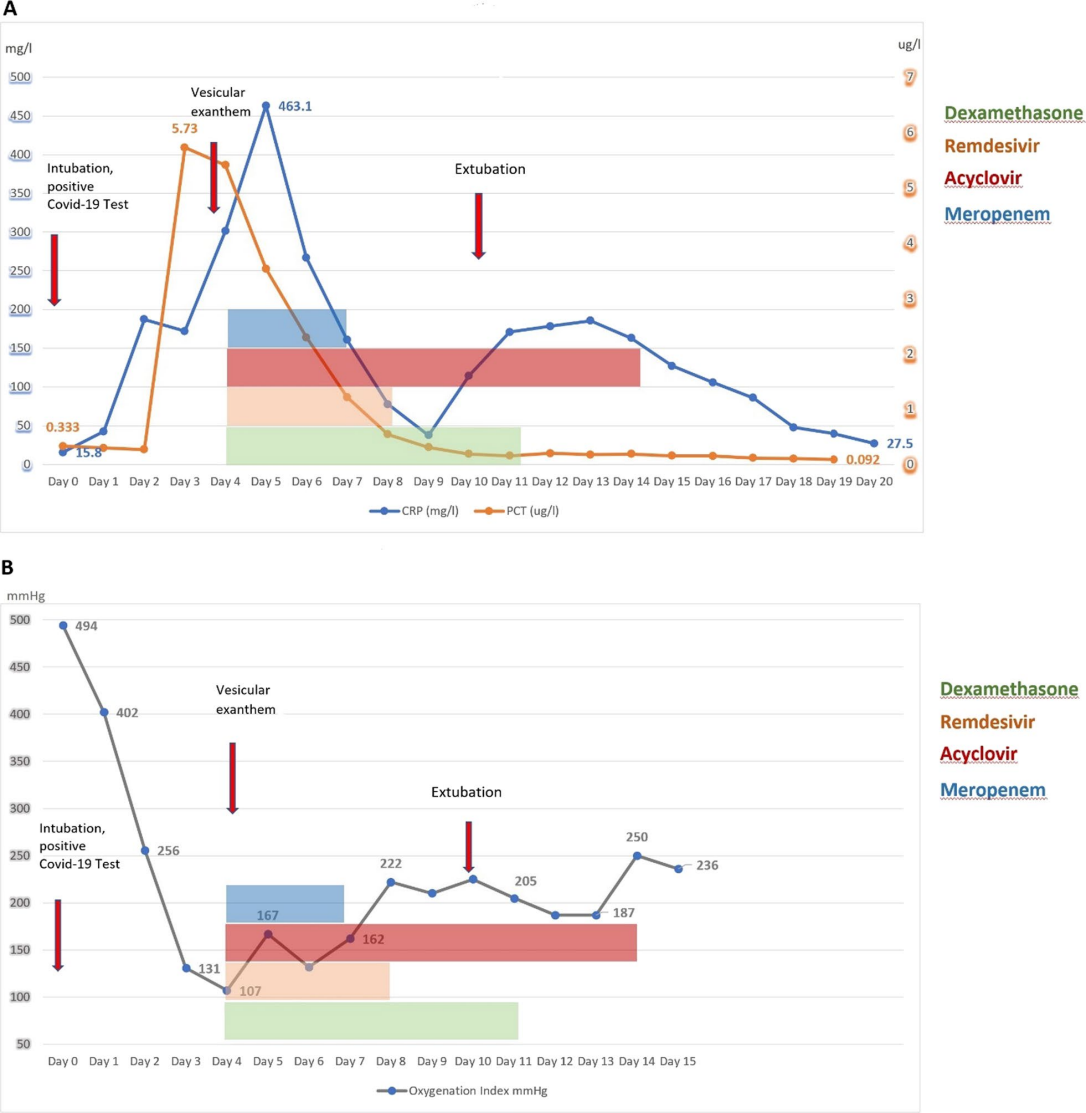
Evolution of **A** C-reactive protein (CRP) and procalcitonin (PCT) and **B** the oxygenation index over the course of diagnostics and treatment

We observed a fast pulmonary and hemodynamic stabilization. Continuously decreasing values of C-reactive protein and procalcitonin along with a good neurological and pulmonary recovery (Fig. 3a, b) allowed for extubation 11 days after admission to the ICU and transfer to the normal ward after 20 days. After 46 days of hospitalization, the patient was discharged and transferred to a rehabilitation clinic.

## Discussion

SARS-CoV-2 spreads mainly via respiratory droplets and has an incubation period of up to 14 days. The most common symptoms of COVID-19 are fever, cough, fatigue, gastrointestinal disorder, hyposmia, and hypogeusia. However, a wide range of other symptoms have been reported such as neurological and cardiovascular manifestations.

Skin lesions have been reported in up to 20% of patients hospitalized for COVID 19 [6]. The most typical cutaneous manifestations including dengue-like exanthema, plaques on the heels, and urticaria have been associated with prognosis, severity, and stadium of the SARS-CoV-2 infection [7].

Recalcati described a chickenpox-like rash in one patient hospitalized for COVID-19 [8]. A chickenpox-like rash with a median latency period of 3 days and a median duration of 8 days is being considered a rare but specific manifestation of COVID-19 [9]. Vesicular lesions seem to appear mostly in the early stages of COVID-19 in middle-aged patients and are usually associated with an intermediate severity of the disease. On the other hand, the potential immunosuppressant effect of COVID-19 could not only facilitate the development of possible coinfections but also have an influence on their severity. *In vitro* studies suggest that SARS-CoV-2 could affect the quantity and the function of cluster of differentiation(CD)4+ and CD8+ T cells, and natural killer cells [3, 5]. It could also affect the synthesis and functionality of interferons 1 and 2, which lead to higher viral loads [2, 4]. Both mechanisms may lead to significant immunosuppression. Bacterial coinfections and superinfections have been described in up to 28% of patients with COVID-19 admitted to the ICU [10]. They have a great prognostic and therapeutic impact, and early implementation of empiric antibiotic therapy is often crucial in case of progressive pulmonary deterioration. Viral coinfections, in contrast, appear to be less common. Lansbury *et al*. reported viral coinfections in 3% of patients with COVID-19 [11]. The possible role of SARS-CoV-2 in the reactivation of viral infections such as VZV and herpes simplex virus has been suggested previously [12, 13]. In the clinical case described here, the cause of the skin lesions was a primary VZV infection, not a reactivation, which is a rare condition in adulthood, representing only 5% of all varicella cases. Nevertheless, mortality in adults is responsible for up to 55% of all varicella-related deaths [14]. Similar average incubation periods of COVID-19 and varicella as well as the timing of clinical manifestations in our patient lead us to the conclusion that VZV and COVID-19 were in our case community-acquired coinfections.

Compared with children, adult patients are 25 times more likely to develop varicella-related complications, of which varicella pneumonia is the most frequent. Pregnancy, history of pulmonary disease, and smoking, as well as a compromised immune system, render patients more susceptible to pulmonary complications of varicella [15]. Varicella pneumonia is associated with a mortality of around 10% in adult patients, which almost doubles in the absence of antiviral treatment [16]. Varicella pneumonia usually becomes symptomatic 1–6 days after the onset of the rash, presenting with signs of massive respiratory distress. Acyclovir reduces mortality and should be initiated early in patients with suspected or diagnosed varicella-pneumonia [16]. In this case, it may be postulated that a compromised immune system due to several factors including trauma and operation but also as a consequence of the COVID-19 might have made our patient prone to pulmonary complications of varicella. Therefore, the onset of unusual skin lesions should raise suspicion of a concomitant infectious process, prior to interpreting the entire picture as COVID-19 related.

This clinical case of an adult patient with COVID-19 and a primary VZV coinfection with possible pulmonary involvement and a polymicrobial ventilator-associated tracheobronchitis highlights the importance of considering coinfections and superinfections in patients with COVID-19 with unusual clinical manifestations.

## Conclusion

A possible immunosuppressant effect of SARS-CoV-2 [5] could increase the likelihood of coinfections and influence their severity in patients presenting with COVID-19. Despite the great variety of possible clinical manifestations of COVID-19, a coincidence of rare symptoms and fast cardiopulmonary deterioration should raise suspicion of a concomitant infectious process. This case of an adult presenting with SARS-CoV-2 infection and simultaneous primary VZV infection illustrates the importance of considering coinfections in patients with COVID-19 with unusual clinical manifestations.

## Data Availability

The datasets used and analyzed during the current study are available from the corresponding author on reasonable request. The clinical data are stored electronically in the intensive care clinical information system software (MetaVision, iMDsoft; ISMed eKG, Protecdata AG) of the University Hospital Basel.

## Abbreviations

ARDS: Acute respiratory distress syndrome
COVID-19: Coronavirus disease 2019
CRP: C-reactive protein
CT: Computed tomography
FiO_2_: Fractional concentration of inspired oxygen
GCS: Glasgow coma scale
ICP: Intracranial pressure
ICU: Intensive care unit
PCR: Polymerase chain reaction
PCT: Procalcitonin
PEEP: Positive end expiratory pressure
VZV: Varicella zoster virus
WHO: World Health Organization
